# See-through optical combiner for augmented reality head-mounted display: index-matched anisotropic crystal lens

**DOI:** 10.1038/s41598-017-03117-w

**Published:** 2017-06-05

**Authors:** Jong-Young Hong, Chang-Kun Lee, Seungjae Lee, Byounghyo Lee, Dongheon Yoo, Changwon Jang, Jonghyun Kim, Jinsoo Jeong, Byoungho Lee

**Affiliations:** School of Electrical and Computer Engineering, Seoul National University, Gwanak-Gu Gwanakro 1, Seoul, 08826 South Korea

## Abstract

A novel see-through optical device to combine the real world and the virtual image is proposed which is called an index-matched anisotropic crystal lens (IMACL). The convex lens made of anisotropic crystal is enveloped with the isotropic material having same refractive index with the extraordinary refractive index of the anisotropic crystal. This optical device functions as the transparent glass or lens according to the polarization state of the incident light. With the novel optical property, IMACL can be utilized in the see-through near eye display, or head-mounted display for augmented reality. The optical property of the proposed optical device is analyzed and aberration by the anisotropic property of the index-matched anisotropic crystal lens is described with the simulation. The concept of the head-mounted display using IMACL is introduced and various optical performances such as field of view, form factor and transmittance are analyzed. The prototype is implemented to verify the proposed system and experimental results show the mixture between the virtual image and real world scene.

## Introduction

Head-mounted display for virtual reality (VR-HMD) and augmented reality (AR-HMD) have gained a lot of popularity in the recent commercial market. VR-HMD uses the floating lens to image panel located near eye at the desired depth. With the large field of view (FOV), large eyebox, and the simplicity of the structure, the VR-HMD has been commercialized, recently^1, 2^. Given success of VR-HMD on commercialization, there have been a lot of approaches to realize AR-HMD^3–14^. Since the AR-HMD should show the real world scene as well as the virtual information, AR-HMD requires optical combiner to combine the virtual information with the real world scene. Among them, the beam splitter (BS) is widely used in the AR application^3–8^. The AR-HMD using BS as optical combiner can provide large FOV, but the form factor of the system is bulky due to the BS. In order to make compact system with BS, there are several approaches such as convex half mirror and free-form optic^4, 5^.

There have been several systems using diffractive optical elements (DOEs) with waveguide as optical combiner. Since the DOEs can control the propagation direction of the light with the diffraction effect on the grating, the form factor of the system can be enhanced. However, in this system, the field of view (FOV) is limited by the structural limitation due to the high-order noise and color separation^10, 11^.

In this paper, we propose the novel optical combiner, index-matched anisotropic crystal lens (IMACL) and the AR-HMD system which can achieve the large FOV. The lens made of the anisotropic crystal is enveloped with the isotropic material having same curvature of the anisotropic crystal lens. When the refractive index of the isotropic material is same for a certain polarization state of the anisotropic crystal lens, the IMACL functions as a transparent glass or a lens according to the polarization state of the incident light. Using this multi-functional property of the IMACL, we demonstrate the practical HMD application using the IMACL. The IMACL is located in front of the human eye and the transparent screen is located in line with the IMACL. The virtual image is polarized to the polarization state of lens mode in IMACL and the real world image is polarized to the polarization state of the glass mode in IMACL.

There have been various researches utilizing the anisotropic crystal to improve the ability of the three-dimensional (3D) display or HMD^6, 15–18^. In addition, there have also been studies to utilize the polarization selectivity by matching the refractive index of certain polarization axis^19–22^. However, to the best of our knowledge, it is the first time to utilize the index-matched anisotropic crystal lens to realize the see-through near eye display, especially AR-HMD.

The AR-HMD system using IMACL has the significance not only because it is an initiative study with the novel structure but also because the FOV and the form factor can be enhanced by the proposed system compared with the conventional AR-HMD system. Especially, the system using the IMACL has the structure similar to the VR-HMD, and the proposed system has the possibility of large FOV. In addition, since the IMACL is located in line with the human eye and transparent screen, the system using IMACL has a large eyebox and it does not have aberrations caused by off-axis system using half convex lens and free-form optics. Furthermore, the system using IMACL can be a promising candidate with the development of transparent flat panel like transparent OLED because of the similarity of the structure with the VR-HMD.

## Results

### Index-matched anisotropic crystal lens

#### Basic configuration of head-mounted system using index-matched anisotropic crystal lens

**Figure 1:**
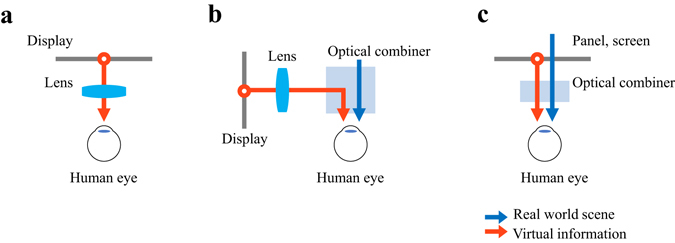
Basic configurations of various HMDs: (**a**) VR-HMD, (**b**) conventional AR-HMD, and (**c**) AR-HMD with IMACL.

Figure 1(a) shows the concept diagram of the VR-HMD. The floating lens is placed in front of the human eye and the display located near eye is floated to the desired depth. The focal length and the lens aperture decide the FOV and the form factor of the system^23, 24^ so the small focal length and large lens aperture are recommended. Figure 1(b) represents the conventional AR-HMDs including the systems using BS and DOE. The display module is located at the side part of the observer and the displayed virtual information is bent to the human eye by the optical combiner. The floating lens is usually located in front of display module as shown in Fig. 1(b), so the system has the bulky form factor.

If the AR-HMD has a structure similar to the VR-HMD, as shown in the Fig. 1(c), the compact system with large FOV can be realized. In order to implement the system shown in the Fig. 1(c), the transparent display or screen is required and the multi-function optical combiner is needed, operating as lens and glass simultaneously.

#### Basic concept of index-matched anisotropic crystal lens

**Figure 2:**
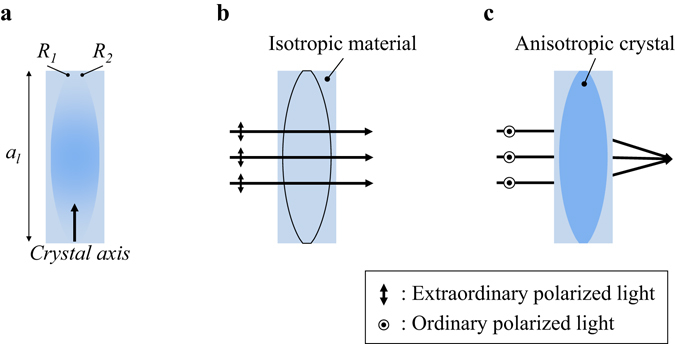
The principle of the IMACL: (**a**) the basic structure of the IMACL, (**b**) when the input polarization is matched to the extraordinary axis of the anisotropic crystal lens (see-through mode), and (**c**) when the input polarization is matched to the ordinary axis of the anisotropic crystal lens (lens mode).

The IMACL is proposed as the multi-function optical element working as glass and lens at the same time. The basic concept of the IMACL is presented in Fig. 2. The anisotropic crystal lens is enveloped with the isotropic material which has the same curvature of the anisotropic crystal lens. The refractive index of the isotropic material is matched with the smaller value of the refractive indices of the anisotropic crystal. In case of the positive anisotropic crystal, the isotropic material should have the same refractive index with the ordinary refractive index of the anisotropic crystal, and in case of the negative anisotropic crystal, the isotropic material should have the same refractive index with the extraordinary refractive index of the anisotropic crystal.

The Fig. 2(a) represents the basic structure of the IMACL. The negative anisotropic crystal is assumed and the *R*_1_ and *R*_2_ are the curvature radii of the anisotropic crystal lens and *a*_*l*_ is the aperture size of the anisotropic crystal lens. To show the functionality of the IMACL, the incident light is assumed as parallel rays. The extraordinary-polarized incident rays go through the IMACL without refraction as shown in Fig. 2(b) because the refractive indices of isotropic and anisotropic material are same in the extraordinary-polarized light. Meanwhile, the IMACL operates as a lens to the ordinary-polarized incident rays, so the incident rays are focused at the focal plane of the IMACL as shown in Fig. 2(c). When the positive anisotropic crystal is used as lens, the basic functionality is same with the Fig. 2, but the polarization state of the incident light is reversed compared with the case of the Fig. 2.

In fabrication step of the anisotropic crystal lens, orientation of crystal axis decides the refractive index of each polarization state of anisotropic crystal. The optical axis of the anisotropic crystal should be perpendicular to the primary direction of the incident light so that the birefringent property of the IMACL is maximized.

The focal length of IMACL is decided by lens curvature and the index difference as follows:

1 $$\frac{1}{f}={n}_{i}(\frac{{n}_{a}}{{n}_{i}}-1)(\frac{1}{{R}_{1}}-\frac{1}{{R}_{2}}+\frac{({n}_{a}-{n}_{i})d}{{n}_{a}{R}_{1}{R}_{2}}),$$

where *n*_*a*_ and *n*_*i*_ are refractive indices of the anisotropic crystal in lens mode and refractive index of the isotropic medium, respectively, and *d* is the thickness of the lens. As presented in Eq. (1), focal length of the IMACL is decided by the index difference between the two polarization states of the anisotropic crystal. Therefore, anisotropic crystal having large index difference is recommended to make short focal length of IMACL, because the short focal length of lens guarantees the small form factor as shown in the Fig. 1.

#### Aberration analysis of index-matched anisotropic crystal lens

Since the AR-HMD system using IMACL merges the virtual information with real world scene in line, there is little aberration from off-axis configuration. However, since the IMACL is made of the anisotropic crystal, aberration is caused by the anisotropy. Especially, in case of the birefringent crystal, refractive index is different according to the angle between the crystal axis and the coordinate, so the astigmatism is the dominant aberration factor^25^. The refractive index of anisotropic material is generally indicated by index ellipsoid and in index ellipsoid, it is well known that the extraordinary refractive index varies depending on the direction of light, while the ordinary refractive index has the same refractive index regardless of the direction of the light. Thus, when the crystal axis of the anisotropic crystal lens is set to the *y*-direction, the focal length in the *x*-direction and the focal length in the *y*-direction become different. There have been studies that show the index deviation induces the astigmatism of the anisotropic crystal lens^25, 26^. The astigmatism due to index deviation in extraordinary refractive index is represented in different types in IMACL according to the kind of anisotropic crystal. The IMACL with positive anisotropic crystal uses extraordinary-polarized state as lens, so the distortion is represented in lens imaging relation. On the other hands, the IMACL with negative anisotropic crystal uses extraordinary-polarized state as transparent glass, so the distortion is represented in see-through mode. To analyze the aberrations in IMACL, the IMACL is simulated with the CODE V^27^.

**Figure 3:**
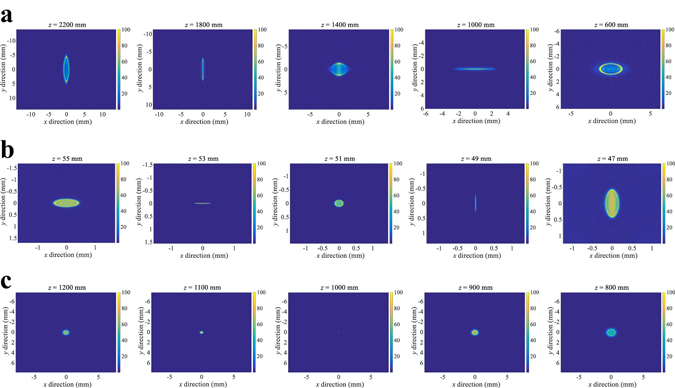
The aberration analysis of IMACL: (**a**) shows the PSF of the IMACL with positive anisotropic crystal when the point light source is located 55 mm behind the IMACL. (**b**) and (**c**) show the PSF of the IMACL with negative anisotropic crystal where the point light source is located 50 mm and 1000 mm behind the IMACL, respectively.

The Fig. 3 shows the point spread function (PSF) of the IMACL. Figure 3(a) shows the PSF of the IMACL with positive anisotropic crystal and Fig. 3(b) and (c) show the PSF of the IMACL with negative anisotropic crystal. The refractive indices of each polarization state are 1.2 and 1.6, respectively, and the radius of curvature is 45 mm. Hence, the effective focal length of the lens is formed near the 58 mm. In Fig. 3(a), a point light source is located 55 mm behind the IMACL and the PSF is measured in lens mode because the IMACL with positive anisotropic crystal uses the extraordinary-polarized state as lens mode. Figure 3(a) shows the PSF variation along the *z*-direction and it is shown that the focal plane of the *x*-direction is formed at the depth of 1000 mm and focal plane of *y*-direction is formed at the depth of 1800 mm. Since the anisotropic crystal in IMACL is birefringent material, the index deviation by the anisotropic property is only represented in *y*-coordinate and it induces the astigmatism of the lens. The distance between focal plane of *x*-direction and *y*-direction is 800 mm which is considerable value in imaging system.

Figure 3(b) and (c) show the simulation results of IMACL with negative anisotropic crystal lens. In this case, the IMACL operates as glass in extraordinary-polarized state, so the see-through mode is simulated. In Fig. 3(b), the point light source is located 50 mm behind the IMACL and in Fig. 3(c), the point light source is located 1000 mm behind the IMACL. The distance between the plane that focuses in the *x*-direction and the plane that focuses in the *y*-direction is only 4 mm when the point light source is located 50 mm behind the IMACL. On the other hand, when the point light source is located far from the IMACL about 1000 mm, the astigmatism and the depth distortion are negligible. In the simulation specification, the variation of the incident angle to the IMACL is about 20 ° where the point light source is located 55 mm from the IMACL and it causes the index deviation of 0.068. However, in case that the point light source is located far from the IMACL, the variation of the incident angle is small and the index deviation rarely affects the image formation. In the simulation specification where the point light source is located 1000 mm behind the IMACL, the index deviation is only 0.00002.

If the point light source is located far from the IMACL, the IMACL with positive anisotropic crystal also has little astigmatism. However, since we focus on the applicability of IMACL in AR-HMD, the IMACL with positive anisotropic crystal is simulated in condition where the virtual image is formed in long distance.

Therefore, IMACL has the small distortion when the image in the long distances is observed through the IMACL with the negative anisotropic crystal. Because the real world of interest is located at more than 500 mm, astigmatism caused by index deviation can be ignored. Hence, based on the analysis, it can be deduced that the negative anisotropic crystal is more suitable for implementing IMACL.

### Augmented reality head-mounted display using index-matched anisotropic crystal lens

IMACL can be utilized in various systems due to its novel property that two optical functions can be performed at the same time by the polarization multiplexing. Especially, IMACL can improve various optical performance of the HMD with its own characteristic.

**Figure 4:**
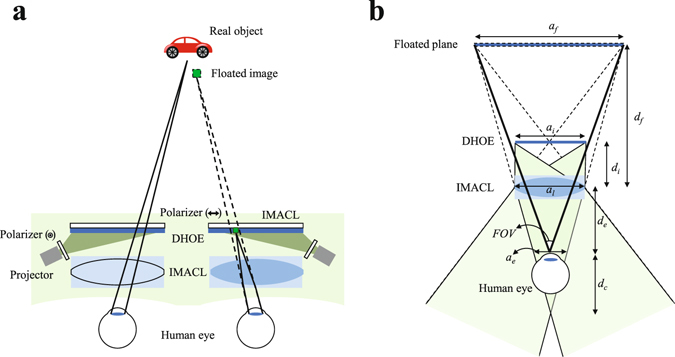
Schematic diagram of AR-HMD system using IMACL: (**a**) left eye shows transparent mode of the IMACL and right eye shows the lens mode of the IMACL, and (**b**) the principle of the FOV of AR-HMD system using IMACL.

Figure 4 shows the basic configuration of the proposed AR-HMD system using IMACL. As shown in left part of the Fig. 4(a), rays from the real object are polarized by the extraordinary polarizer located behind the transparent diffuser. The extraordinary-polarized rays from the real object are not refracted by the IMACL, so the see-through real world information can be observed. Meanwhile, the rays from projector are ordinary-polarized by the polarizer and diffused by the transparent diffuser. The diffused ordinary-polarized light is focused by the IMACL and user can observe the floated image. As a transparent diffuser, diffuser holographic optical element (DHOE), index-matched diffuser, and scattering polarizer can be good candidates^28–31^. Among them, since the DHOE shows real world scene with high transmittance due to its angular selectivity, we adopt the DHOE as transparent diffuser in this scheme.

As shown in the Fig. 4(b), the FOV and the form factor of the AR-HMD system using IMACL are decided by various factors such as focal length of the IMACL (*f*), aperture size of the IMACL (*a*_*l*_), eye relief (*d*_*e*_), eyebox (*a*_*e*_) and desired floating distance (*d*_*f*_). In general, the form factor of the AR-HMD system is related to the projection module and the thickness of the optical combiner. In this paper, the thickness of the optical combiner does not refer to the thickness of the IMACL itself, but to the distance between IMACL and the DHOE which is the minimum distance for the IMACL to float image to desired depth. Since the proposed system succeeds the structure of the VR-HMD, the FOV definition of the system is similar with that of the VR-HMD. When the eye relief and the eyebox are given by the target specification, the virtual information area (*a*_*f*_) is decided and the FOV of the system is also decided as shown in the Fig. 4(b). The FOV is presented as follows:

2 $${d}_{c}=\frac{{d}_{e}{a}_{e}}{{a}_{l}-{a}_{e}},$$

3 $${a}_{f}=\frac{{a}_{e}({d}_{c}+{d}_{e}+{d}_{f})}{{d}_{c}},$$

4 $$FOV=2\,{\tan }^{-1}(\frac{{a}_{f}}{2({d}_{f}+{d}_{e})}).$$

The size of the display should be designed to cover full FOV which is decided in Eq. (4). The size of the display is deducted by the lens makers’ law as presented in Eq. (6).

5 $${d}_{i}=\frac{{d}_{f}f}{f+{d}_{f}},$$

6 $${a}_{i}=\frac{{d}_{i}{a}_{f}}{{d}_{f}},$$

where *d*_*i*_ is distance between the DHOE and the IMACL (thickness of optical combiner) and *a*_*i*_ is size of the display.

**Figure 5:**
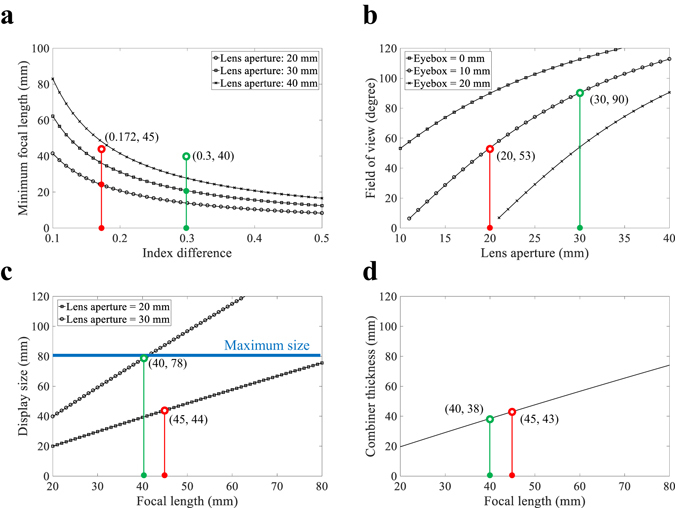
System performance of the AR-HMD system using IMACL: (**a**) Minimum focal length according to the index difference and lens aperture, (**b**) FOV according to the aperture size and eyebox, (**c**) required display size to have maximum FOV according to the focal length and lens aperture, and (**d**) the thickness of combiner according to the focal length.

Figure 5 visualizes the key factors of the proposed AR-HMD such as FOV, thickness of combiner and required display size based on above equations. The eye relief (*d*_*e*_) of 10 mm and the floating depth (*d*_*f*_) of 1000 mm are assumed. Figure 5(a) shows the minimum focal length according to the lens aperture and index difference. As shown in the Fig. 5(a), high index difference is required to implement IMACL having short focal length. The minimum focal length represents the focal length of the IMACL when the radius of curvature of the IMACL is half of the lens aperture like ball lens. Therefore, as shown in Fig. 5(a), since large lens aperture induces large radius of curvature, minimum focal length becomes small. However, in the case of ball lens with minimum focal length, it is necessary to design a lens with a larger radius of curvature in practice, because it distorts the image severely due to aberrations. Figure 5(b) shows the relationship between lens aperture and FOV for various eyebox based on Eq. (4). As shown in Eq. (4), the FOV of the proposed system is determined by the lens aperture and the eyebox. The FOV is in trade-off relation with the eyebox, and the large lens aperture guarantees wide FOV. The eyebox is area where the image from display can be seen without loss when the human eye is placed on the eye relief as shown in Fig. 4(b). Therefore, when the eyebox of the system is set wide, the FOV decreases because the active area of the display which can provide the image to the human eye becomes narrow. Figure 5(c) and (d) show the size of the display required to obtain the full FOV and the distance between the IMACL and the DHOE required to float the image at the desired depth according to the focal length of the IMACL. The display size presented in Fig. 5(c) is the value required to obtain the maximum FOV. If the display size becomes smaller than this value, FOV of the system becomes lower. The maximum size line (blue line) in Fig. 5(c) represents the limit of the display size required not to overlap with the display for the other eye. Figure 5(d) shows the thickness of the combiner according to the focal length. In general, for an AR-HMD that reproduces images at a distance of 1 m or more, the combiner thickness has a value similar to the focal length.

Therefore, at a given eye relief and desired floating depth, the FOV of the proposed system is in a trade-off relationship with the eye box and increases as the size of the lens aperture increases. Meanwhile, under certain lens apertures, the focal length of IMACL is determined by the index difference. The focal length determines the display size and the thickness of optical combiner that are related to the form factor of the system. Thus, short focal length is required to achieve small display size and optical combiner thickness. Therefore, the performance of the proposed system is maximized when IMACL has large lens aperture and short focal length, which are obtained from anisotropic crystal with high index difference. Thus, the proposed system requires the anisotropic crystal with high index difference.

The red point and the green point are examples of the proposed system. The red point is an example used as a test bed in this paper and the green point is an example of ideal case. For the red point, we assumed an IMACL with a lens aperture of 20 mm and a focal length of 45 mm at index difference of 0.17. This allows the system to have FOV of 90 ° when the eyebox is 0 mm and FOV of 53 ° when the eyebox is 10 mm. The display size required to implement the system with eyebox of 10 mm and FOV of 53 ° is 44 mm and the thickness of the optical combiner is 43 mm. For the green point, we assumed an IMACL with a lens aperture of 30 mm and a focal length of 40 mm at index difference of 0.3. This allows the system with FOV of 112 ° when the eyebox is 0 mm and FOV of 90 ° when the eyebox is 10 mm. The display size required to implement the system with eyebox of 10 mm and FOV of 78 ° is 38 mm and the thickness of the optical combiner is 43 mm.

The transmittance of the whole system is decided by the transmittance of the polarizer, the DHOE, and the IMACL. Among those, the DHOE has high transmittance over 0.9 and IMACL also has the transmittance over 0.8 to the polarized incident light, so the AR-HMD system using IMACL can provide bright real world scene.

### Experimental setup and results

#### Implementation of IMACL

The IMACL is fabricated with calcite (CaCO_3_) in our experiment which is the representative negative anisotropic crystal. There are various birefringent materials of high index difference including liquid crystal and titanium dioxide (TiO_2_) from 0.3 to 0.4, but in this paper, we use the calcite for verification of the proposed concept, which is easy to fabricate. The calcite is immersed in the index matching liquid to implement the IMACL and the refractive index of index matching liquid is 1.486 which is equal to the extraordinary refractive index of the calcite.

As following the Eq. (1), the focal length of the IMACL is calculated to be 42 mm and the measured focal length of IMACL is 45 mm. The detailed specification of fabricated IMACL is presented in Table 1.

**Table 1:** Specification of implemented IMACL.

Lens specification	Value
Diameter (*a*_*l*_)	20 mm
Radius of curvature (*R*_1_, *R*_2_)	14.98 mm
Thickness of the lens (*d*)	10.64 mm
Extraordinary refractive index of lens (*n*_*e*_)	1.486
Ordinary refractive index of lens (*n*_*o*_)	1.658
Refractive index of isotropic material	1.486

#### See-through head-mounted display using index-matched anisotropic crystal lens

**Figure 6:**
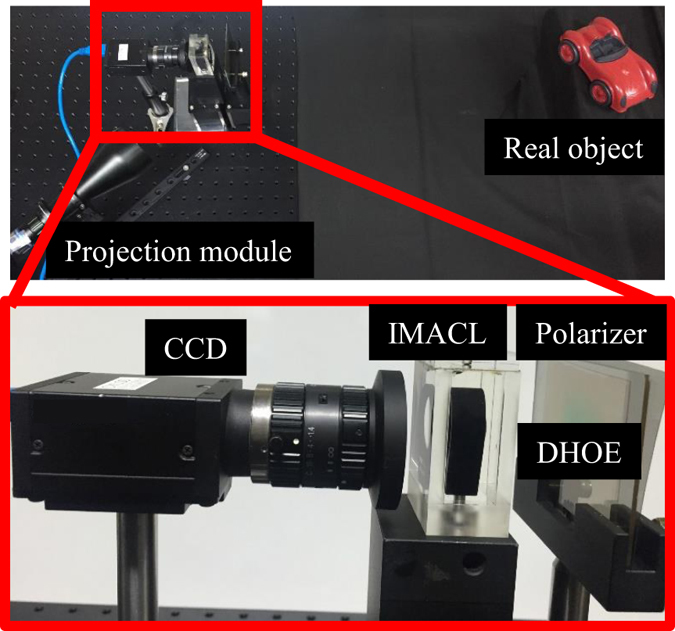
Experimental setup of proposed AR-HMD system using IMACL.

Figure 6 represents the experimental setup of the proposed see-through HMD display. As a transparent screen, the DHOE is adopted and light from the projector is collimated to coincide the reconstruction condition with the recording condition. The detailed specification of the experimental setup is presented in Table 2.

**Table 2:** Specification of proposed AR-HMD system using IMACL.

System specification	Value
Resolution of the projector	1920 (H) × 1080 (V) mm
Thickness of the optical combiner	43 mm
Display(DHOE) size (eyebox of 0 mm)	87 mm (required), 50 mm (experiments)
Display(DHOE) size (eyebox of 10 mm)	44 mm (required), 50 mm (experiments)
FOV (eyebox of 0 mm)	90 ° (achievable), 60 ° (experiments)
FOV (eyebox of 10 mm)	53 ° (achievable), 53 ° (experiments)
Transmittance	31%

**Figure 7:**
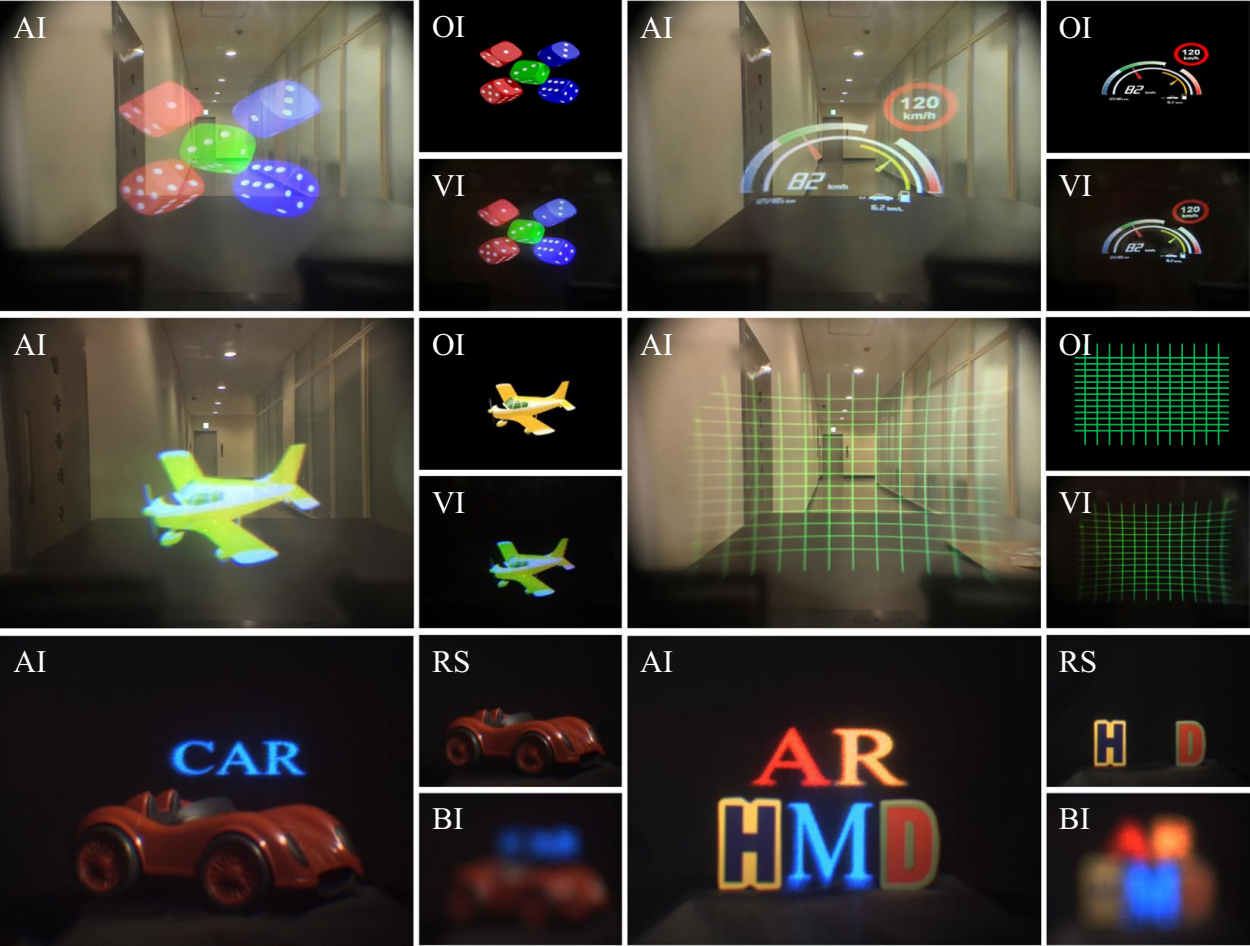
Experimental results of proposed AR-HMD system using IMACL. The dashboard, dices, stripes and planes are captured by the cell phone camera (Supplementary Video 1). The augmented reality image (AI) is shown and the original image (OI) and the virtual image (VI) only are also shown. The letters are captured by the CCD. The real world scene (RS) and the blurred image (BI) are shown. The rendered airplane image has free license for personal and commercial use and publicly available on the website: http://www.cadnav.com/3d-models/model-28772.html. The rendered dices image is under the Creative Commons Attribution-Share Alike 3.0 Unported license (https://creativecommons.org/licenses/by-sa/3.0/deed.en) and publicly available on the website: https://en.wikipedia.org/wiki/File:PNG_transparency_demonstration_1.png.

The distance between IMACL and DHOE is about 43 mm, so the projected image on the DHOE is floated on the 1000 mm away from the IMACL. Figure 7 shows the visualization results with see-through HMD display using IMACL. The dashboard, dices, stripes, and plane are augmented to the real object. The images are floated on the plane of the real object which is 1000 mm away from the observer. The four upper images with dices, dashboard, plane and stripes are captured by the camera (iPhone 7, Apple) and the camera is located in eye relief (10 mm). The video is also presented to show the feasibility of our prototype (Supplementary Video 1). With the DHOE and the IMACL, the full color virtual information is reconstructed. To show the floated image clearly, we show the virtual information (VI) by blocking the real world scene as well as the merged image. Two bottom images with letters are captured by the charge-coupled device (CCD) with lens of narrow depth of field to show the position of the virtual information. The real objects (red car, letters ‘H’ and ‘D’) are located in 1000 mm away from IMACL. The virtual information and the real object have almost same blurring effect and they are focused identically.

As shown in Fig. 5(c), the display size is important to provide the maximum FOV of the proposed system. The display size depends on the size of the projected image and the size of DHOE. In our laboratory environment, the maximum recording size of the DHOE is about 50 mm, and the maximum size of projected image from the projector is also about 50 mm. Since Fig. 7 is captured at a fixed camera position (eyebox of 0 mm), the display size for FOV of 90 ° should be at least 87 mm. However, as the display size and projected area are limited to 50 mm, the FOV of our prototype is limited to 60 °. On the other hand, the maximum FOV is 53 ° with required DHOE size of 43 mm when the eyebox is 10 mm. In other words, our experimental prototype has the limitation to show the maximum FOV when the camera is fixed (eyebox of 0 mm), but it has enough display size for the eyebox of 10 mm.

As presented above, since the AR-HMD system using IMACL is similar with the VR-HMD, the IMACL will become worthy of notice with the development of the transparent display. The concept of the flat panel type AR-HMD system is also proposed and the feasibility of the system is shown in Supplementary document.

## Discussion

The novel optical combiner which is called IMACL to realize the AR-HMD has been proposed. The IMACL is made of the anisotropic crystal lens enveloped with the isotropic material. Hence, the IMACL can be a multi-function optical element according to the polarization state. Because of the property of the IMACL, the IMACL can be located in line with the human eye as optical combiner. Therefore, we have proposed novel AR-HMD structure using IMACL. With the proposed AR-HMD system, the large FOV can be realized. We have verified the proposed idea with the prototype. The calcite is used as anisotropic crystal and the FOV of the prototype reaches up to 60. It is expected that the study about AR-HMD system using IMACL will be adopted significantly to have large FOV and small form factor.

## Methods

### Diffuser holographic optical element

**Figure 8:**
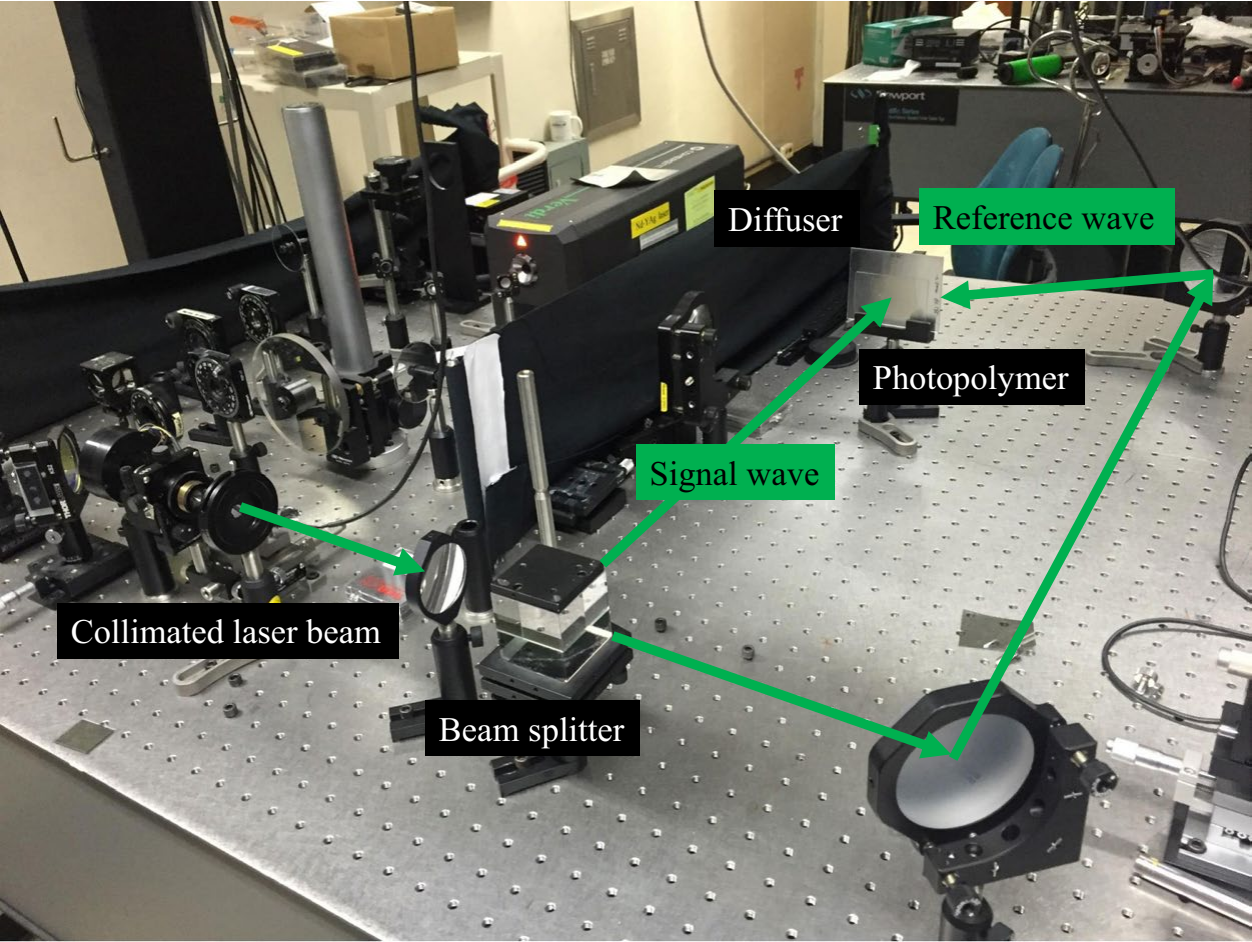
Recording experimental setup of DHOE.

The DHOE provides an image in the proposed system as a transparent screen without losing transparency^30, 31^. A photopolymer (HX film provided from Covestro AG) is used for holographic material and red (Cobolt Flamenco, 660 nm), green (Coherent Verdi, 532 nm), and blue (Spectra-Physics Excelsior, 473 nm) lasers are used to realize the full color DHOE with the wavelength multiplexing. The recording setup of the DHOE is presented in Fig. 8.

**Figure 9:**
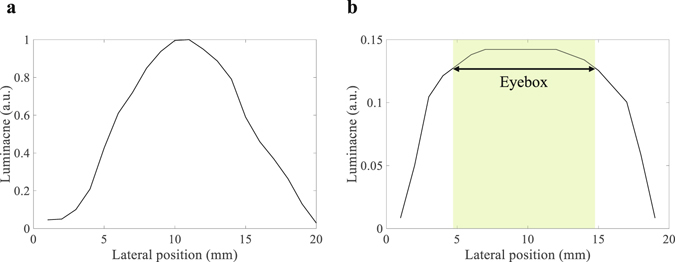
Measured luminance distribution of (**a**) DHOE with diffusing angle of 10 ° and (**b**) DHOE with diffusing angle of 60 °.

In our prototype, the diffusing angle of 26 ° is required for light from DHOE at a distance of 43 mm to cover lens aperture of 20 mm. Also, the DHOE of 44 mm is needed to provide system with eyebox of 10 mm and FOV of 53 °, and in this case, diffusing light at the edge of the DHOE requires a diffusing angle of about 62 ° to cover the whole eyebox. Therefore, we record DHOE using a diffuser with a diffusing angle of 60 °. To verify the effect of diffusing angle of DHOE on the performance of the system, we record DHOEs with diffusing angle of 10 ° and 60 °. The luminance distributions of each diffuser are measured at the position of the eye relief in the proposed system using luminance meter (Konika Minolta, CA-210) as shown in Fig. 9.

The luminance of this system is measured by moving a luminance meter with interval of 1 mm. The aperture size of the luminance meter is 1 mm and a white image is projected on the DHOE. The DHOE with diffusing angle of 10 ° shows nonuniform luminance distribution because the diffusing light from the DHOE does not sufficiently cover the lens aperture shown in Fig. 9(a). On the other hand, as shown in Fig. 9(b), the DHOE with a diffusing angle of 60 ° can obtain uniform luminance distribution. Luminance varies less than 10 percent within the eyebox of 10 mm which is target specification of prototype. The DHOE with diffusing angle of 60 ° provides uniform luminance distribution, but it has lower intensity of diffusing light than that of DHOE of 10 ° due to its wide diffusing angle. However, in general, since the intensity of the projector is bright enough, the it is sufficient to provide augmented virtual information to real world scene using the DHOE with diffusing angle of 60 °.

### Transmittance of optical devices

**Figure 10:**
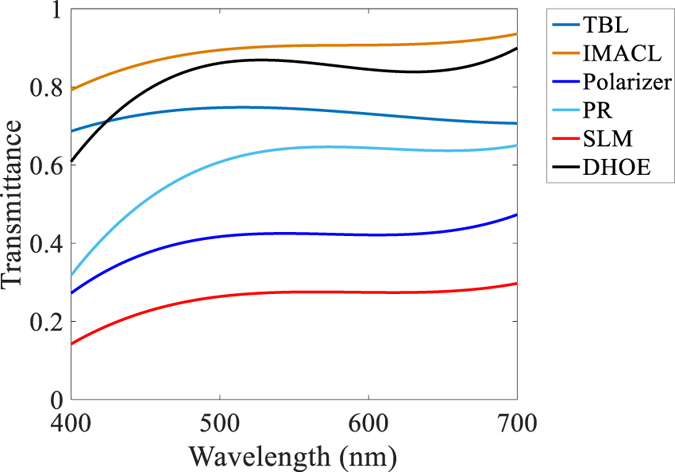
Transmittance of the optical devices.

The transmittance of the optical devices is measured with the spectrometer (Oceanoptics, USB4000-VIS-NIR-ES) as presented in Fig. 10. Figure 10 includes the transmittance of the optical devices of flat panel type AR-HMD system in Supplementary document as well as prototype in this paper. The moving average values of transmittance are presented for clear recognition. TBL is the transparent backlight, and PR is polarization rotator. The transmittance of the IMACL is measured for the polarized light and one polarizer of the SLM is detached because the polarization rotator has linear polarizer in it.

## Electronic supplementary material

Supplementary video 1

Supplementary information
