# Supplementary material for: See-through optical combiner for augmented reality head-mounted display: index-matched anisotropic crystal lens
Source: Sci Rep. 2017 Jun 5;7:2753. doi: 10.1038/s41598-017-03117-w (PMC5459829; doi:10.1038/s41598-017-03117-w)
Supplement: Supplementary file 2 — Supplementary information [file 41598_2017_3117_MOESM2_ESM.pdf]

Supplementary information

See-through optical combiner for augmented reality head-mounted display: index-matched anisotropic crystal lens: supplementary material

Jong-Young Hong, Chang-Kun Lee, Seungjae Lee, Byounghyo Lee, Dongheon Yoo, Changwon Jang, Jonghyun Kim, Jinsoo Jeong, and Byoungho Lee[*]

School of Electrical and Computer Engineering, Seoul National University, Gwanak-Gu Gwanakro 1, Seoul 08826, South Korea

*Corresponding Author's E-mail: Byoungho@snu.ac.kr

Part 1. Supplementary Video 1: Visualization of proposed system using IMACL

Visualization result of proposed AR-HMD prototype using IMACL. Moving dices are captured with cell phone (iPhone 7, Apple). The rendered dices image is under the Creative Commons Attribution-Share Alike 3.0 Unported license (https://creativecommons.org/licenses/by-sa/3.0/deed.en) and publicly available on the website: https://en.wikipedia.org/wiki/File:PNG_transparency_demonstration_1.png.

Part 2. Flat panel type AR-HMD system using IMACL

In this section, we show the concept of the flat panel type AR-HMD using the IMACL. As improving the transparency of the flat panel, the proposed system using IMACL can be a good candidate for ultimate AR-HMD. However, in current stage, the flat panel does not guarantee the enough transparency, so we show concept and feasibility of the system. Flat panel type AR-HMD can have an advantage of lightweight and compact system because it does not require the projection distance and projection module. However, since it is hard to separate the virtual information on the flat panel and the real world scene, there are little approaches to realize the flat panel type AR-HMD[1].

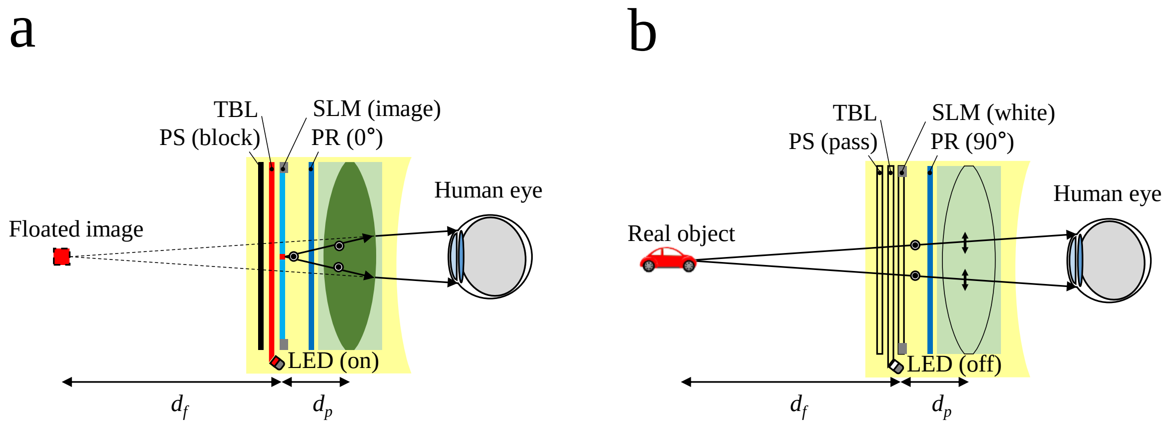

Figure S1. Schematic diagram of the proposed flat panel type AR-HMD: (a) image frame and (b) see-through frame

Figure S1(a) and (b) show the schematic diagram of the proposed flat panel type AR-HMD. The system is composed of IMACL, polarization rotator (PR), polarization switch (PS), LC panel, and the transparent backlight (TBL). The polarization rotator located in front of the IMACL divides the frame to the see-through frame and the lens frame. At the see-through frame in the Fig. S1(a), the polarization rotator rotates the incident light to the extraordinary-polarized light and at the lens frame, it rotates the incident light to the ordinary-polarized light. Hence, at the see-through frame, the LC panel provides the white image to transmit the light and extraordinary-polarized transmitted light by the polarization rotator goes through the IMACL without any refraction. Meanwhile, at the lens frame, the LC panel provides the virtual information which is floated to the infinity or near the real object by the IMACL because the polarization rotator makes the polarization state of the light from the LC panel to the ordinary polarization state. The polarization switcher located in front of the SLM is extraordinary polarizer to show the real world scene clearly at the see-through frame and ordinary polarizer to block the outside information at the lens frame. With this time-multiplexing method, the real world scene and the virtual information is combined. In the lens mode of the IMACL by the polarization rotator, the SLM shows the image set of each color channel. Images for red, green and blue is divided and displayed at each frame. In the glass mode of the IMACL by the polarization rotator, the SLM shows the white image to transmit the incident light from real world scene. With the time-multiplexing technique, these four frames are merged and makes full color image. In case of monochrome realization, the monochrome image and the white image are alternately displayed. The full color realization sacrifices the image frame of 25% and monochrome realization sacrifices the image frame of 50%.

The TBL is implemented with the transparent diffuser similar with the transparent anisotropic diffuser (TAD) and convex-half-mirror array (CHMA)[2,3]. As a transparent diffuser, the TBL transmits the real world scene and reflects the light emitting diodes (LEDs) light.

The floated plane of the virtual image (d_f) is decided by the focal length of the IMACL (*f*) and the distance between the lens and LC panel (d_p) as presented in Eq. (S1).

$$d_f = \frac{f d_p}{d_p - f}. \quad \text{(S1)}$$

As presented in Eq. S1, the image is floated to the infinity at the focal length of the IMACL and so, the focal length decides the form factor. When the focal length of the IMACL is short, the distance between display and the IMACL can decrease and the size of the display to cover full FOV also decreases and it increases the compactness of the system.

The resolution of the system is decided by the resolution of the flat panel. However, in current stage, the flat panel consists of the pixel structure which induces the diffraction effect and it makes the resolution limitation[1]. The LC panel is transparent basically, but the black matrix of the LC panel decreases the transparency and incident light from the real world scene is diffracted by the pixel structure. The diffraction effect becomes severe as smaller pitch of the pixel structure has and it degrades the see-through property. Therefore, the flat panel type AR-HMD has the resolution limitation with the pixel structure LC panel.

The transmittance is also crucial point of the AR-HMD. We define the transmittance to the ratio of how much does incident light from the real world scene to the observer. The transmittance of the flat panel type system is limited by the transmittance of each device (SLM, polarization rotator, polarization switch, and IMACL). The IMACL operates in certain polarization state, so the incident light should be polarized and it reduces the transmittance by 50%. In addition, we use the time-multiplexing technique, the transmittance decrease by multiplexing ratio.

The time-multiplexing and the resolution limit of the pixel structure may hinder the sufficient AR experience in current stage. However, since every optical component including the display module is located in front of the eye in-line, the flat panel type AR-HMD using IMACL has compact system configuration. Also, the proposed flat panel type AR-HMD system can be utilized more with the development of the transparent flat panel technology like transparent OLED.

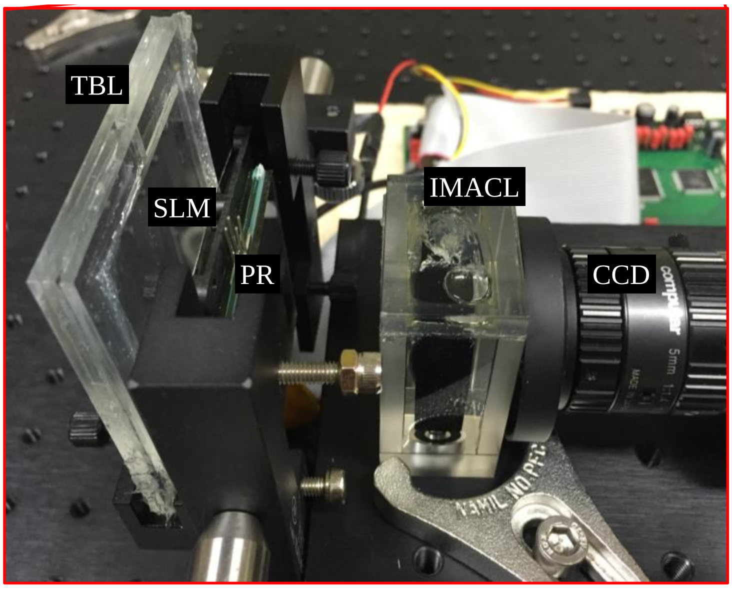

Figure S2. Experimental setup of proposed flat panel based AR-HMD system

Figure S2 shows the configuration of the prototype. To show the high resolution floated image, the monochrome SLM (LCX-017, Sony) is used in our preliminary prototype. The detailed specification is as follows in Table S1.

Table S1. Specification of proposed flat panel based AR-HMD system

Specification	Value
Panel resolution	1024 (H) × 768 (V)
Panel pixel pitch	32 μm
Distance between IMACL and panel	40 mm
Distance between real object and IMACL	357 mm
FOV	52°
Framerate	28 Hz
Transmittance	5%

As shown in the Fig. S2, the TBL which consists of waveguide and metal coated index matched diffuser is located behind the system. IMACL is located 40 mm in front of the SLM to float the

image in the 350 mm away, Figure S3 shows the experimental results. The dashboard image which are virtually overlapped in the real world scene are floated. The framerate of the display system is measured about 28fps and transmittance of the system is measured to 0.05. The PR, SLM, and LED is synchronized using the PR controller, Arduino, and Vesa video signal. As shown in the Fig. S3, the transmittance of the real world scene decrease.

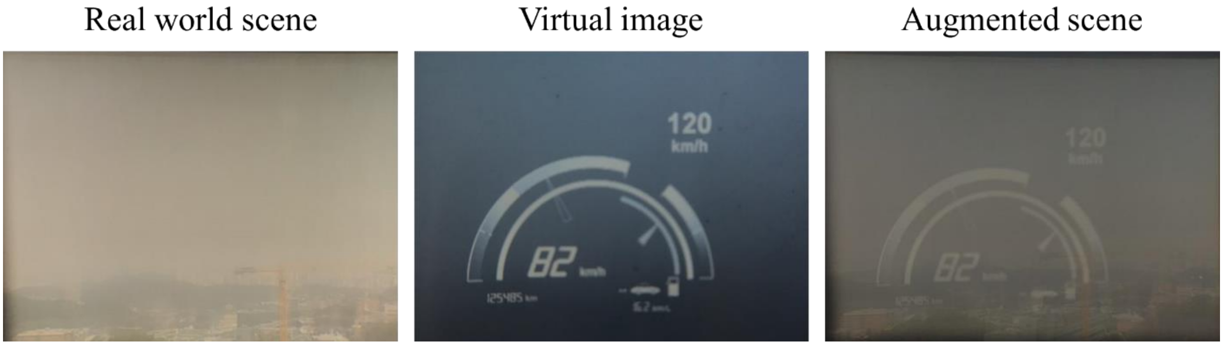

Figure S3. Experimental results of proposed flat panel based AR-HMD system

Reference

1. A. Maimone, D. Lanman, K. Rathinavel, K. Keller, D. Luebke, and H. Fuchs, "Pinlight displays: wide field of view augmented reality eyeglasses using defocused point light sources," in "ACM SIGGRAPH 2014 Emerging Technologies," (ACM, 2014), p. 20.

2. J. Hong, Y. Kim, S.-g. Park, J.-H. Hong, S.-W. Min, S.-D. Lee, and B. Lee, "3d/2d convertible projection-type integral imaging using concave half mirror array," Optics Express 18, 20628–20637 (2010).

3. J.-Y. Hong, S.-G. Park, C.-K. Lee, S. Moon, S.-J. Kim, J. Hong, Y. Kim, and B. Lee, "See-through multi-projection three-dimensional display using transparent anisotropic diffuser," Optics Express 24, 14138–14151 (2016).
